# Cellular Transplantation for Liver Diseases

**DOI:** 10.4021/gr2008.11.1243

**Published:** 2008-11-20

**Authors:** Elizabeth Jameson

**Affiliations:** Department of Medicine, McGill University, 3655 Promenade Sir William Osler, Montreal, Quebec, Canada, H3G 1Y6. Email: elizabeth.jameson@elf.mcgill.ca

**Keywords:** cell transplantation, liver failure, stem cells, orthotropic liver transplantation

## Abstract

Presently, the orthotropic liver transplantation (OLT) is still the most effective therapeutic for patients with acute or chronic hepatic failure. However, due to the shortage of donor livers, the number of patients benefited from this approach is limited. Therefore, some alternative modalities have been paid attention for restoring the liver function. The cell transplantation is one of the promising modalities to realize this purpose. The types of cells used in the cell transplantation include syngeneic hepatocytes, allogeneic hepatocytes, immortalized hepatocytes, and stem cells derived heptocytes. The stem cells, especially the adult stem cells from bone marrow, are shown as a promising cell source for liver repopulation. The mesenchymal bone marrow stem cells and embryonic stem cells can be induced to differentiate into the hepatic lineage and might be used in the cell transplantation for liver diseases. Compared to OLT, the advantages of cell-based therapy for liver disease are, but not limited to, less invasive, less expensive, easy manipulated, easy expansion of cells in vitro. Cells can be stored in a cell bank for future use. Though most of the current studies are experimental and animal based, the cellular therapy for liver disease is expected to be an effective alternative in clinical settings in near future.

## Introduction

Up to now, orthotropic liver transplantation (OLT) is still the most effective treatment of a variety of life-threatening liver diseases, however, due to the shortage of donors, the number of patients who can benefit from this modality is very limited(1, 2). In recent years, cell based therapies have been investigated as alternatives to whole liver transplantation. There are some advantages of cellular transplantation over whole liver transplant. A single donor liver could be shared by multiple patients; it is less invasive, procedures are safer and easier to manage, multiple treatments can be carried out on a single patient(3); only 1-5 percent of the total hepatocytes mass transplantation could correct a variety of metabolic disorders(1); the hepatocytes can be manipulated in vitro easily, such as genetic manipulation(3, 4). This brief review summarizes some highlights in the cellular transplantation in the treatment of liver diseases, such as the source of cells, routes of transplantation, and indications of transplantation.

## Potential clinical applications of cellular transplantation for liver diseases

### Bridging patients with liver failure to whole liver transplantation

This is to improve the patient’s condition and extend short-term survival until a donor liver becomes available or until the patient’s own liver recovers(3, 5, 6). Previous clinical observations using liver cell transplantation for the hepatic failure showed that the patients’ condition was transiently improved and thus successfully bridged to OLT(5, 7).

### Correct of inborn errors of metabolism

The studies of cellular transplantations for inborn errors of metabolism have been conducted both in animal models(8-14) and in humans(15-18). These animal models with genetic liver defects include Gunn rat, which lacks hepatic bilirubin UDP-glucuronosyl transferase actificty (UGT1A1), and the Nagase analbuminemic rat model(19, 20). Allogeneic hepatocytes transplantation was used as a treatment for inherited glycogen storage type 1a(21) and the crigler-Najjar syndrome type I(16).

### Liver failure and cirrhosis

When patients with acute liver failure were transplanted allogeneic hepatocytes, clinical improvement was observed though the donor hepatocytes constitute approximately 1% to 10% of the liver mass. However, there was no survival improvement in the extremely sick patients(6).

The causes of liver disease often involve exposure to extrinsic agents, especially drugs or environmental toxins, which induce acute to subacute hepatocellular disease(3). The thearapeutic significance of hepatocytes transplantation is likely to be disease context-dependent. In the D-galactosamine model, there was increased survival after transplantation of hepatocytes subcellular fractions, culture supernatants or even bone marrow cells, these results indicate that the early therapeutic effect requires only cell-derived factors(22-24) for the first 3-5 days after induction of acute liver failure. After this period, the transplanted hepatocytes will repopulate and proliferate with reorganization to restore liver function, and reproduce fully normal hepatic tissue, this process needs several weeks or months(3), thus, it is of significance for the treatment of chronic liver disease, such as liver cirrhosis(25).

## Types of cells for transplantation in liver diseases

### Primary hepatocytes

The adult primary hepatocytes were used commonly in experimental and pre-clinical studies(26). These cells include allogeneic, xenogeneic or autologus primary isolated hepatocytes. Primary xenogenic hepatocytes seems to be an unlimited source of hepatocytes and can solve the human donor shortage(27-30). When the porcine hepatocytes transplantated into a rat cirrhotic model, it can correct liver function(29). However, using xenogenic hepatocytes transplantation may incur some concerns, such as xenozoonoses, xenoantifenicity(31), and immunorejection, host versus graft reaction(32).

### Immortalized cell lines

The immortalized heaptocytes are derived from gene transfer(32-48) in vitro. The conditionally immortalized rat hepatocytes by transduction using the simian virus 40 T antigen(SV40 Tag) can lower the risk of transplanted hepatocyte malignant transformation(49), because after excision of the SV40Tag, these immortalized hepatocytes can stop growing and the differentiated characteristics are enhanced(50). These conditionally immortalized cell lines transplantation in rats with end-stage liver failure can provide life-supporting liver-specific metabolic function(49). When immortalized heaptocytes isolated from P19ARF null mice were intrasplenically transplanted into Fas-injured liver of SCID mice (severe combined immunodeficiency), the cells participated the liver repopulating and regenerating process, and were capable of generating hepatic progenitor cells during liver restoration(51).

### Hepatocyte-like cells derived from adult bone marrow stem cells

Previous studies showed that bone marrow cells contains a subpopulation of hepatocytes-orientated stem cells expressing a-AFP, c-met, CD34 and c-kit(31, 52, 53). These cells in vitro and in vivo can differentiated into hepatocytes(54-56) with the capacity of reversing liver failure(56), and synthesizing albumin in the lethally irradiated mice(57).

### Hepatocyte-like cells derived from embryonic stem cells

The embryonic stem cells can differentiate into hepatocytes in vitro(58-60) and in vivo(61, 62). The embryonic stem cells could differentiate into hepatocytes under specific culture conditions. Mouse embryonic stem cells were able to grow and showed morphology consistent with typical mature hepatocytes and expressed hepatocytes specific genes, when these cells transplanted into the carbon tetrachloride-injured mouse liver, they were able to incorporated into liver tissue and rescued mice from hepatic injury(63). The embryonic stem cells derived hepatocytes have been found to be effective in a liver failure mouse model induced by diphtheria toxin(64).

## Routes of cell transplantation

### Intraperitoneal injection

The peritoneal cavity is a commonly used site for cell implantation(65). The peritoneal cavity has a relatively large space into which a remarkable number of cells can be transplanted.

### Intrahepatic transplantion

The intrahepatic transplantation of normal hepatocytes can prevent Wilson's disease in these LEC rats(66). In another study, the intrahepatic transplantation of human hepatocytes in immunodeficient, fumarylacetoacetate hydrolase-deficient (fah(-/-) mice, three months after transplantation, about 20% of the mouse liver was observed repopulated by human hepatocytes, and sustained expression of lentiviral vector transduced gene(67).

### Portal veinous infusion

Hepatocytes can be seeded into the liver by portal vein infusion and the transplanted hepatocytes can integrate into the liver cords, leaving the hepatic architecture intact(68). Hepatocytes engrafted in the liver have the benefits of exposure to the portal nutrients, contacting with other hepatocytes and nonparenchymal cells, thus have the ability to secrete bile into the indigenous biliary system (33).

### Intrasplenic infusion

Intrasplenic transplantation is another route of cell delivery for the liver diseases. Previous studies showed that intrasplenic transplantation of hepatocytes increased survival of the totally hepatectomized rats(69). The intrasplenic transplantation of human fetal immortal hepatocytes could prolong survival of 90% hepatectomized rats(70). After massive hepatectomy, such as in 90% PH, or under the situation that the diseased liver is not a suitable site for cell transplantation, the intrasplenic cell transplantation may become an ideal alternative. Hepatocytes can be injected into the splenic pulp from which most cells translocate to the liver through the splenic vein, a small fraction of the cells engraft within the spleen and can develop into bile cannuliculae, sinusoidal structures, and endothelial cells, producing a similar structure to the liver(71, 72).

## Storage and availability of cells

Isolated hepatocytes can be stored in a cryopreserved hepatocytes bank. Since human liver cells are sensitive to damage due to the freezing-thawing procedure, reliable procedures for long-term cryopreservation of human hepatocytes need to be investigated(1). The major concern of using heaptocytes is the lack of donor availability, in addition, the cryopreserved liver cells have not yet been shown to engraft as well as fresh hepatocytes(73).

## Immunorejection

To solve the shortage of donor livers, researchers turn to the cells from animal sources, the animal source seems to be an unlimited supply. However, the xeno-transplantation can cause host immunorejection and the immunosuppressive agents must be used(10, 74).

## Considerations for future studies

Though some positive results obtained in the cell based therapy for liver disease either in animal or clinical studies, there are, however, still some important theoretical and practical issues that need to be addressed. Firstly, whatever transplantation route employed, the cells engraftment and cell efficacy are crucial points(75), the functions of transplanted cells are closely related to the engraftment rate. The use of better quality cells and regeneration stimulus can improve the engraftment(76, 77). Secondly, if stem cell derived hepatocytes are used, these cells may have potential of malignant transformation. Thirdly, except the autologus or syngeneous cell sources, the allogeneic or xenogeneic cell transplantation needs co-administration of immunosuppressive agents which may aggravate or contraindicate the patients’ illness.

## Conclusions

Cellular therapy is a promising approach for the liver diseases, although there are a number of issues unsolved. With the cellular and molecular biology advancement, it is hopeful that cell transplantation will be developed into a standard therapy for liver failure or inborn metabolic diseases in the future.
